# Dissociation Between Progressive Retinal Nerve Fibre Layer Thinning and Perimetric Change in Multiple Sclerosis-Associated Uveitis

**DOI:** 10.3390/jcm15218376

**Published:** 2026-10-27

**Authors:** Tomislav Vidović, Josip Knežević, Sonja Jandroković, Sanja Perić, Jelena Metikoš, Dina Lešin Gačina, Marija Bukvić, Tamara Mišljenović Vučerić

**Affiliations:** 1 Department of Ophthalmology, School of Medicine, University of Zagreb, Clinical Hospital Center Zagreb, 10000 Zagreb, Croatia; josipkn@yahoo.com (J.K.); sonja.jandrokovic@mef.hr (S.J.); sanja.peric@kbc-zagreb.hr (S.P.); dina.lesin@kbc-zagreb.hr (D.L.G.); marija.bukvic2@kbc-zagreb.hr (M.B.); 2 Department of Ophthalmology, Clinical Hospital Center Osijek, 31000 Osijek, Croatia; jelena.metikoss@gmail.com; 3 Department of Ophthalmology, Clinical Hospital Center Rijeka, 51000 Rijeka, Croatia; tamara.misljenovic@ri.t-com.hr; 4 TIM oftalmologija, 51000 Rijeka, Croatia

**Keywords:** multiple sclerosis, uveitis, intermediate uveitis, optical coherence tomography, retinal nerve fibre layer, perimetry, asymptomatic

## Abstract

**Background and Objective**: Uveitis occurs more frequently in multiple sclerosis (MS) than in the general population and is often clinically silent. We determined the anatomical types and visual field defect patterns of MS-associated uveitis, and compared the 24-month course of peripapillary retinal nerve fibre layer (pRNFL) thickness and perimetric mean defect (MD) between symptomatic and asymptomatic patients. **Methods**: Retrospective longitudinal study of 32 MS patients with uveitis diagnosed by SUN criteria, none with prior optic neuritis. Cirrus HD-OCT and Octopus 900 N1 perimetry were performed at three annual visits. Linear mixed models accounted for inter-eye correlation; annualized rates of change were calculated. **Results**: Uveitis was identified in 32 of 852 referred patients (3.8%). Intermediate uveitis predominated (59.4%). Fourteen patients (43.8%) were asymptomatic, yet visual field defects were present at baseline in 84.4% of the full cohort, despite generally preserved visual acuity. pRNFL declined in every subgroup (*p* < 0.0001), including non-inflamed fellow eyes. Within the bilateral uveitis subgroup (*n* = 24), thinning was faster in asymptomatic than symptomatic patients (−2.75 vs. −1.40 µm/year; Mann–Whitney *p* = 0.033), persisting in direction and magnitude after exclusion of three patients with cystoid macular oedema, although significance in that restricted sample was test-dependent (Mann–Whitney *p* = 0.054); the corresponding MD difference did not persist (*p* = 0.292). These symptom-based comparisons were exploratory. **Conclusions**: MS-associated uveitis is frequently asymptomatic but accompanied by subclinical perimetric loss, supporting examination irrespective of symptoms. pRNFL thinning was not confined to inflamed eyes; without an MS-without-uveitis or healthy comparator its origin cannot be attributed to a single mechanism. Structural and functional change dissociated.

## 1. Introduction

Multiple sclerosis (MS) is a chronic inflammatory demyelinating and neurodegenerative disease of the central nervous system (CNS). It is the leading cause of non-traumatic neurological disability in young adults [1]. According to its clinical course, MS is categorised into several subtypes: Clinically Isolated Syndrome, Relapsing-Remitting MS, Secondary Progressive MS, and Primary Progressive MS [2]. The disease can manifest with a wide range of neurological symptoms, including weakness, numbness or tingling, lack of coordination, dizziness, and slurred speech. Ophthalmological manifestations are common in MS. Acute optic neuritis and eye motility disorders are the most common. Acute optic neuritis could be the first sign of multiple sclerosis in 25% of patients with MS. More than half of MS patients had acute optic neuritis during the disease course [3]. Although less common than optic neuritis, uveitis also occurs in patients with MS. The association between MS and intraocular inflammation has been recognised for decades. Uveitis is defined as inflammation of the uveal tract and is classified anatomically into anterior, intermediate, and posterior forms. In patients with MS, intermediate uveitis is the most frequently reported type. The prevalence of uveitis in MS patients ranges from 0.65% to 36.7% [4], which is approximately 10 times higher than in the general population. The wide range reflects differences in study design, case definition and—importantly—in the source population, as estimates derived from referral or specialist cohorts are not directly comparable with those obtained by systematic screening of unselected MS populations. This elevated prevalence is thought to reflect shared genetic risk factors and immunological effector pathways between the two conditions. However, it remains unclear whether uveitis represents a direct manifestation of MS or a coincident disorder. MS-associated uveitis primarily affects young adult females and typically presents as bilateral intermediate uveitis with vision-related complications [5,6]. There is strong evidence that uveitis in MS is related to the underlying immunological pathology, as the retina—although non-myelinated—is a component of the CNS, and the inflammatory processes observed in the retina in MS-associated uveitis are similar to those occurring in myelinated CNS structures [7]. Despite this, visual symptoms in MS remain difficult to attribute, as demyelination, intraocular inflammation and the ocular adverse effects of disease-modifying therapies may all produce overlapping complaints [5]. MS-related uveitis is associated with diverse complications, including cataract, cystoid macular oedema (CME), band keratopathy, glaucoma, retinal detachment, retinoschisis, vitreous haemorrhage, and occlusive vasculitis [8,9]. Optical coherence tomography has become the standard method for quantifying neuroaxonal loss in the anterior visual pathway in MS. Peripapillary retinal nerve fibre layer thickness reflects the axonal content of the optic nerve and is reduced in eyes with previous optic neuritis, but also, to a lesser degree, in eyes with no clinical history of optic neuritis [10]. This subclinical thinning is regarded as a marker of the diffuse neurodegenerative component of MS rather than of focal inflammatory injury [10]. Standard automated perimetry provides the corresponding functional measure, with the mean defect summarising global sensitivity loss. Whether intraocular inflammation contributes to progressive structural and functional loss in eyes without optic neuritis, and whether this differs between symptomatic and clinically silent uveitis, has not been established. A proportion of MS-associated uveitis is clinically silent and may therefore remain undiagnosed in the absence of routine ophthalmological screening. The aim of this study was to determine the anatomical types, symptom profile and visual field defect patterns of uveitis in patients with multiple sclerosis, and to compare the longitudinal course of peripapillary retinal nerve fibre layer thickness and perimetric mean defect over 24 months between symptomatic and asymptomatic patients and in patients with unilateral anterior uveitis, to compare the course of affected and clinically unaffected fellow eyes. We hypothesised that visual field defects and progressive pRNFL thinning would be present in patients without ocular symptoms as well as in symptomatic patients, and that the rate of pRNFL loss would not be confined to inflamed eyes. Restricting the cohort to patients without a history of optic neuritis removes the dominant cause of retinal axonal loss in multiple sclerosis and therefore allows structural and functional change to be followed without that confounder.

## 2. Materials and Methods

This retrospective longitudinal observational study was conducted at the Division of Neuro-Ophthalmology, Department of Ophthalmology, University Hospital Centre Zagreb across seven years, from 1 January 2019 to 31 December 2025. The study was conducted in accordance with the Declaration of Helsinki and approved by the institutional Ethics Committee on 13 August 2026 (class 8.1-26/219-2, Nr 02/013 DU). Informed consent was waived, since this is a retrospective study and anonymised data were used. Reporting follows the STROBE statement for observational studies.

During the study period, 852 patients with multiple sclerosis (MS) were referred for neuro-ophthalmological evaluation. All referred patients underwent a standardised ophthalmological examination comprising best-corrected visual acuity testing, pupillary reaction testing, slit-lamp biomicroscopy, dilated ophthalmoscopy and Goldmann applanation tonometry. Uveitis was identified in 32 patients (3.8%). Inclusion criteria for the patients: multiple sclerosis confirmed by a neurologist according to revised McDonald criteria [11], uveitis diagnosed and classified according to Standardisation of Uveitis Nomenclature (SUN) criteria [12,13] and optical coherence tomography (OCT) and Octopus perimetry in N1 program at three consecutive annual examinations. Exclusion criteria: patients with ophthalmologic lesions leading to impaired visual function, such as retinal detachment, glaucoma, or degenerative chorioretinal changes. Patients with a history of optic neuritis in either eye and patients with refractive error greater than 3 diopters were also excluded. Of these 32 patients, none met any exclusion criterion, and all were included in the analysis. Each patient contributed three examinations at approximately 12-month intervals, giving a total follow-up of 24 months. All 32 patients underwent an identical diagnostic work-up to exclude alternative aetiologies of uveitis, in accordance with published recommendations for the diagnosis of MS-associated intermediate uveitis [9,12]. This comprised serological and laboratory testing for Toxoplasma gondii, Treponema pallidum, Mycobacterium tuberculosis (interferon-γ release assay and/or tuberculin skin test) and herpes viruses; serum angiotensin-converting enzyme; posteroanterior chest radiography; and magnetic resonance imaging of the brain and orbits. Uveitis was classified anatomically as anterior, intermediate or posterior according to SUN criteria. Patients were analysed in two groups determined by laterality, as anterior uveitis was unilateral in all patients whereas intermediate and posterior uveitis were bilateral in all patients. The presence of cystoid macular oedema (CME) was recorded as a binary variable and confirmed on macular OCT. Patients were classified at baseline as symptomatic if they reported ocular complaints (redness, blurred or cloudy vision, floaters, mild pain or photophobia) at presentation, and as asymptomatic if uveitis was detected on examination in the absence of reported ocular symptoms. Baseline symptomatic status was used as a fixed grouping factor; no patient changed category during follow-up. Disease-modifying therapy status was undocumented in the referral records for 15 of 32 patients. For these patients, the referring neurologists were contacted directly and confirmed that none had received fingolimod at any point during the study period, although the full disease-modifying therapy history could not be reconstructed. Fingolimod-associated macular oedema is therefore an unlikely cause of the cystoid macular oedema observed in three patients, but disease-modifying therapy status remains incomplete for 15 patients and could not be entered into the statistical model. Neurological disability was not analysed. Expanded Disability Status Scale (EDSS) scores were not consistently recorded in the referral documentation during the study period and were therefore unavailable for a sufficient proportion of the cohort to permit analysis. Duration of MS at baseline and disease-modifying therapy were extracted from the neurological records where documented.

Each examination began with taking a detailed history, followed by a complete ophthalmological examination. The examination consisted of BCVA, which was measured with Snellen optotypes at 6 metres and converted to logMAR for analysis; biomicroscopic examination of the anterior segment; Goldmann applanation tonometry; and indirect ophthalmoscopy. Optical coherence tomography. Peripapillary retinal nerve fibre layer thickness was measured with Cirrus HD-OCT (Carl Zeiss Meditec, Dublin, CA, USA; software version 9.0.0.281) using the Optic Disc Cube 200 × 200 acquisition protocol. Global average retinal nerve fibre layer thickness was obtained from the 3.46 mm diameter calculation circle automatically centred on the optic disc. Only scans with a signal strength of at least 7 and free of segmentation errors and motion artefacts were included in the analysis. Visual fields were assessed by standard automated white-on-white perimetry using an Octopus 900 perimeter (Haag-Streit AG, Köniz, Switzerland) with the N1 neurological test programme and the Dynamic threshold strategy (EyeSuite Static Perimetry, version 3.1.1). Only examinations with a reliability factor of 15% or less were accepted. If the RF exceeded this value, the test was repeated within one week. Mean defect was recorded as the global index. Values between −2.00 and +2.00 dB were considered normal. These values were extracted from the Octopus printout. On the Octopus platform, mean defect is expressed as the age-corrected deviation from the normative database, such that higher positive values indicate greater sensitivity loss. Visual field printouts were classified into six patterns by a grader masked to clinical diagnosis, patient identity and visit number. Each visit was graded independently, without access to the previous grading of the same eye. Visual field defects were classified into six categories, modified from Nevalainen et al. [14]. The original classification was condensed into a smaller number of categories owing to the sample size by merging related defect patterns. Six categories: 1. Normal finding 2. Central scotoma 3. Depression of retinal sensitivity in the peripheral zone with paracentral scotoma 4. Nerve fibre bundle defect 5. Sector defect; 6. Depression of retinal sensitivity in the peripheral zone. All examinations were performed at the same institution using the same devices and the same software version throughout the study period. Data were extracted from electronic medical records and device printouts by a single investigator and cross-checked against the source documents. Continuous variables are presented as mean ± standard deviation and categorical variables as counts and percentages. The distribution of patient-level annualized rates of change was assessed with the Shapiro–Wilk test and by inspection of normal quantile plots. Rates of mean defect change were normally distributed (all *p* > 0.05), whereas rates of pRNFL change were not (*p* ≤ 0.027). Comparisons of annualized pRNFL rates between symptomatic and asymptomatic patients were performed with the Mann–Whitney U test, which was the primary inferential test for this comparison; the Welch *t* test and its 95% confidence interval are reported alongside as a descriptive estimate of the mean difference and are not treated as confirmatory. These symptom-based comparisons were exploratory: the subgroups were small, several related comparisons were performed, and no adjustment for multiplicity was applied. Because the posterior uveitis subgroup comprised only five patients, no inferential comparison was performed within that subgroup; uveitis type was instead entered as a fixed factor in the mixed model. Baseline comparisons between asymptomatic and symptomatic patients were performed using the independent-samples *t* test for continuous variables and Fisher’s exact test for categorical variables. Analyses of BCVA (logMAR), mean defect and pRNFL thickness were performed with PROC MIXED (SAS version 9.4; SAS Institute Inc., Cary, NC, USA) and are referred to throughout as linear mixed models. In the bilateral uveitis group, each dependent variable was measured in both eyes at three visits. Patient was entered as a random intercept, the two eyes were treated as nested within patient, and the three visits were treated as repeated measures on eye within patient with a first-order autoregressive AR(1) covariance structure. Fixed effects were symptomatic status, eye, visit, uveitis type (intermediate vs. posterior), and the interactions symptomatic status × visit, eye × visit and eye × symptomatic status. Parameters were estimated by restricted maximum likelihood. In the anterior uveitis group, where all patients were symptomatic and inflammation was unilateral, the same structure was used with eye (affected vs. fellow) and visit as fixed effects and their interaction. For both groups, where a fixed effect reached significance, pairwise differences were examined with the Tukey–Kramer adjustment. A *p*-value < 0.05 was considered statistically significant. Two procedures were used to obtain annualized rates of change and are reported separately. For the rate-of-change analysis, linear regression was fitted to the groupmean value at baseline, 12 and 24 months for each eye category, and theslope multiplied by 12 to give an annual rate (μm/year for pRNFL; dB/yearfor mean defect); the coefficient of determination therefore describes themfit of a linear trend to three aggregate time points and does not reflect between-patient variability. For the comparisons in Section 3.7, an annualized rate was calculated for each patient individually and averaged across the two eyes; these patient-level rates were the unit of analysis for all comparisons between symptomatic and asymptomatic patients. Annualized rates were calculated using the actual interval between examinations, derived from the recorded examination dates, rather than nominal 12-month intervals. These analyses were performed using Python (version 3.11, Python Software Foundation, Wilmington, DE, USA).

## 3. Results

### 3.1. Patients

Thirty-two patients with multiple sclerosis and uveitis were followed for 24 months with examinations at approximately 12-month intervals. The cohort comprised 11 men (34.4%) and 21 women (65.6%). Mean age was 34.59 ± 6.01 years (range 22–46); 33.27 ± 6.15 years in men and 35.29 ± 5.97 years in women. Eight patients (25.0%) had anterior uveitis, and 19 (59.4%) had intermediate uveitis. Posterior uveitis was found in 5 patients (15.6%). The two groups were defined by laterality: anterior uveitis was unilateral in all cases, permitting a within-patient fellow-eye comparison, whereas intermediate and posterior uveitis were uniformly bilateral. These are referred to as the anterior uveitis group (*n* = 8) and the bilateral uveitis group (combined intermediate and posterior uveitis) (*n* = 24).

The diagnosis of anterior uveitis was established by the finding of ciliary injection, corneal endothelium precipitate, and cells in the anterior chamber (Tyndall sign). Patients complained of eye redness, photophobia, blurred vision and periocular pain. All were symptomatic at the first examination. The diagnosis of intermediate uveitis was established by the finding of inflammatory cells in the vitreous and exudate above the pars plana. Three patients had cystoid macular oedema confirmed with OCT and FA. The diagnosis of posterior uveitis in the five patients was established based on the presence of active chorioretinal inflammatory lesions, retinal vasculitis (confirmed by fluorescein angiography), and significant inflammatory cells in the vitreous overlaying the retinal lesions, in accordance with the SUN criteria. Fourteen patients (43.8%) were asymptomatic throughout follow-up, and 18 (56.3%) were symptomatic. All asymptomatic patients had intermediate (*n* = 12) or posterior (*n* = 2) uveitis; no asymptomatic patient had anterior uveitis or cystoid macular oedema (CME). All three patients with CME were symptomatic. Asymptomatic and symptomatic patients did not differ in age (35.1 ± 7.0 vs. 34.2 ± 5.3 years, *p* = 0.703) or sex distribution (*p* = 1.000). Distribution of patients by age, symptomatic status and MS duration is presented in Table 1. All patients received topical corticosteroid drops. Patients with anterior uveitis were additionally treated with mydriatics. The three patients with cystoid macular oedema received a single 0.7 mg dexamethasone intravitreal implant in both eyes one month after the baseline visit. No implant was repeated during the 24-month follow-up. All five patients with posterior uveitis additionally received systemic corticosteroid therapy. Ocular and disease-modifying treatment by group is presented in Table 2. Table 2 records any treatment exposure at baseline or initiated during the 24-month follow-up period.

### 3.2. Visual Acuity

Best-corrected visual acuity was preserved in the majority of eyes. In the anterior uveitis group, BCVA in the affected eye was 0.05 ± 0.07 logMAR at baseline and improved to 0.01 ± 0.02 logMAR at 24 months, while fellow-eye BCVA was 0.00 logMAR at every visit. In the bilateral uveitis group, BCVA was 0.00 logMAR at every visit in all asymptomatic patients. In the five patients with posterior uveitis, BCVA changed by ≤0.01 logMAR throughout follow-up. Visual acuity improved in the three patients with CME following intravitreal therapy, from 0.52–1.00 logMAR at baseline to 0.30–0.40 logMAR at 12 months, and remained stable thereafter; it did not normalise, consistent with the absent ellipsoid zone and external limiting membrane at the fovea in all six eyes (Table 3).

### 3.3. Visual Fields Defect

A visual field defect was present in all 16 eyes of the 8 patients with anterior uveitis at every examination. In affected eyes the predominant pattern shifted from peripheral depression with paracentral scotoma (4/8 at baseline, 1/8 at 24 months) towards a nerve fibre bundle defect (2/8 to 5/8), whereas in the fellow eyes the distribution was unchanged at every visit, with a nerve fibre bundle defect in six of eight. In the bilateral uveitis group, 5 of 24 patients (20.8%) had a normal visual field at baseline and 4 (16.7%) at 12 and 24 months; categories were concordant between right and left eye in all 24 patients at every visit. Central scotoma was present in six patients: the three with cystoid macular oedema and three symptomatic patients with posterior uveitis. At baseline, a visual field defect was present in 27 of 32 patients (84.4%) (Table 4).

### 3.4. Anterior Uveitis Group

**Figure 1:**
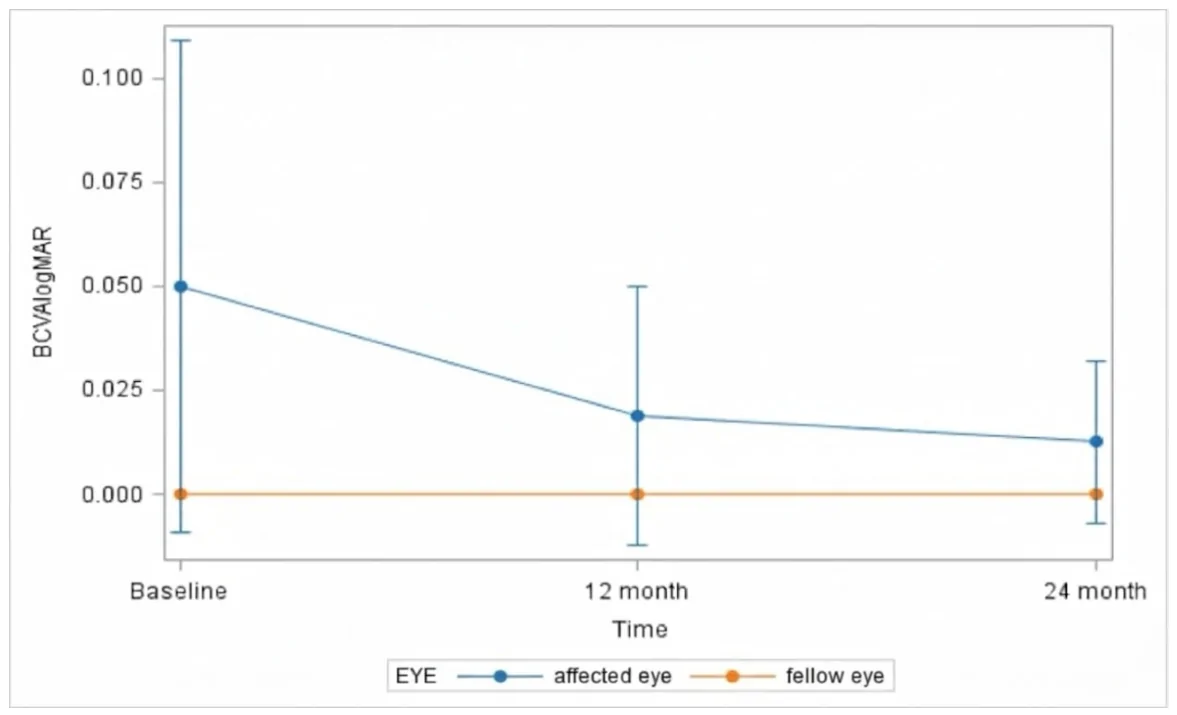
Longitudinal changes in best-corrected visual acuity (BCVA logMAR) over 24 months in the Anterior uveitis group: affected eye and non-inflamed fellow eye. Error bars represent 95% confidence intervals.

**Figure 2:**
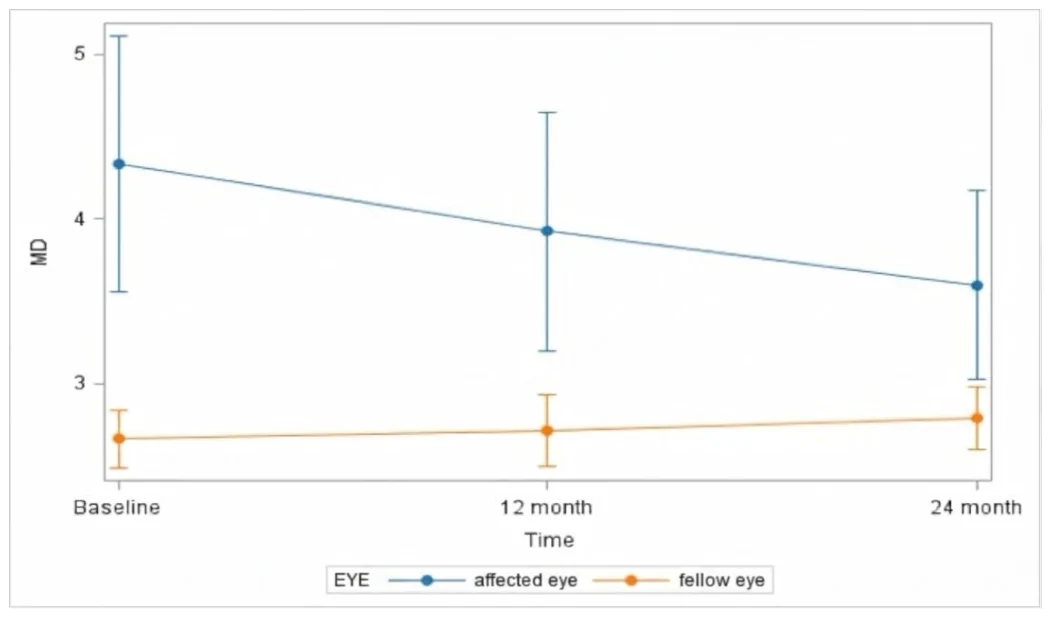
Visual field mean defect (dB) at baseline, 12 and 24 months in the anterior uveitis group: affected eye and non-inflamed fellow eye. Error bars represent 95% confidence intervals.

**Figure 3:**
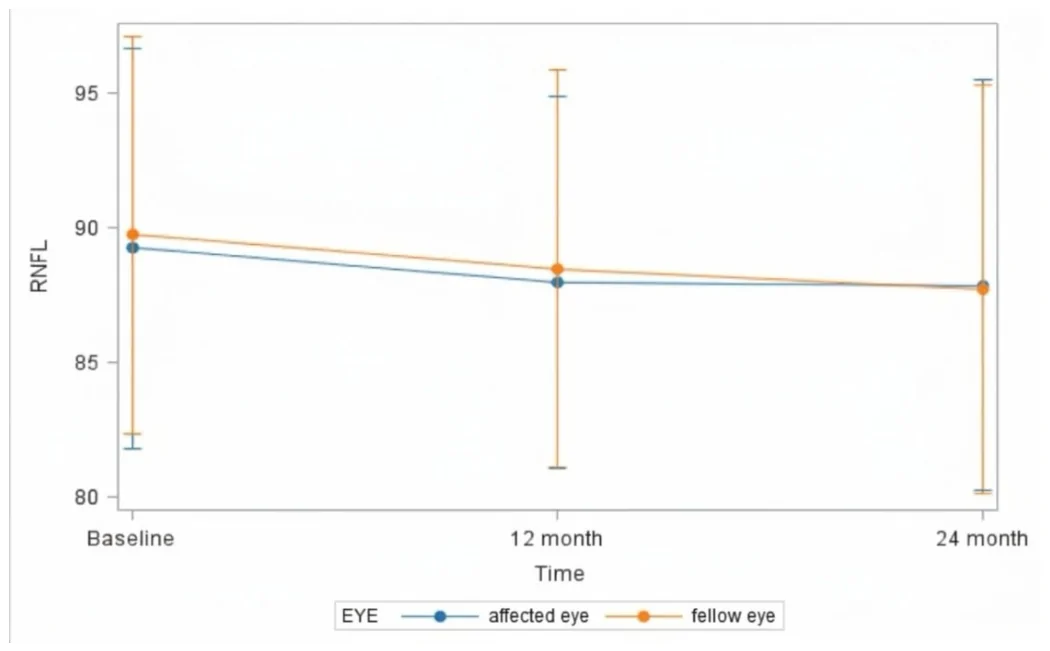
Longitudinal changes in retinal nerve fiber layer (RNFL) thickness over 24 months in the anterior uveitis group: affected eye and non-inflamed fellow eye. Error bars represent 95% confidence intervals.

In the anterior uveitis group, the linear mixed model showed no significant effects for BCVA: eye (*p* = 0.0902), time (*p* = 0.0989) and eye × time (*p* = 0.0989). For visual field mean defect, significant effects were found for eye (F = 24.10, *p* = 0.0014), time (F = 3.64, *p* = 0.0394) and eye × time (F = 7.04, *p* = 0.0033). Mean defect decreased in affected eyes (−0.37 dB/year) and was unchanged in fellow eyes (+0.06 dB/year). For pRNFL thickness, a significant effect of time was found (F = 22.75, *p* < 0.0001), with no significant effect of eye (*p* = 0.1065) or eye × time (*p* = 0.4151); thinning proceeded at −0.69 µm/year in affected and −1.00 µm/year in fellow eyes (Table 5; Figure 1, Figure 2 and Figure 3).

### 3.5. Bilateral Uveitis Group

**Figure 4:**
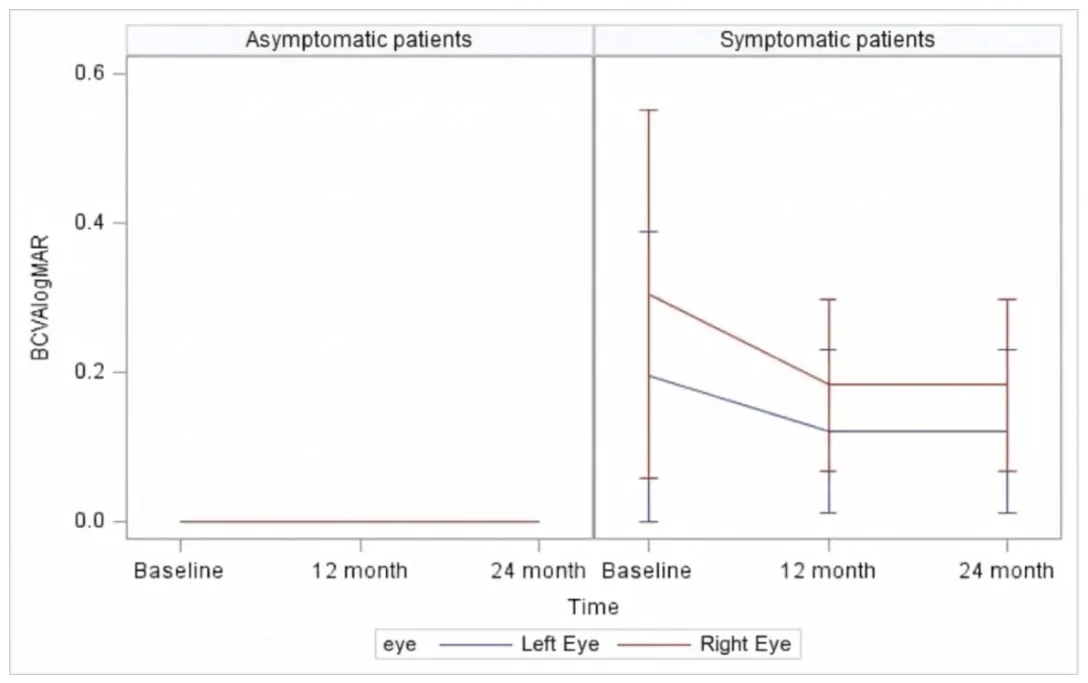
Longitudinal changes in best-corrected visual acuity (BCVA logMAR) over 24 months in the bilateral uveitis group. Error bars represent 95% confidence intervals.

**Figure 5:**
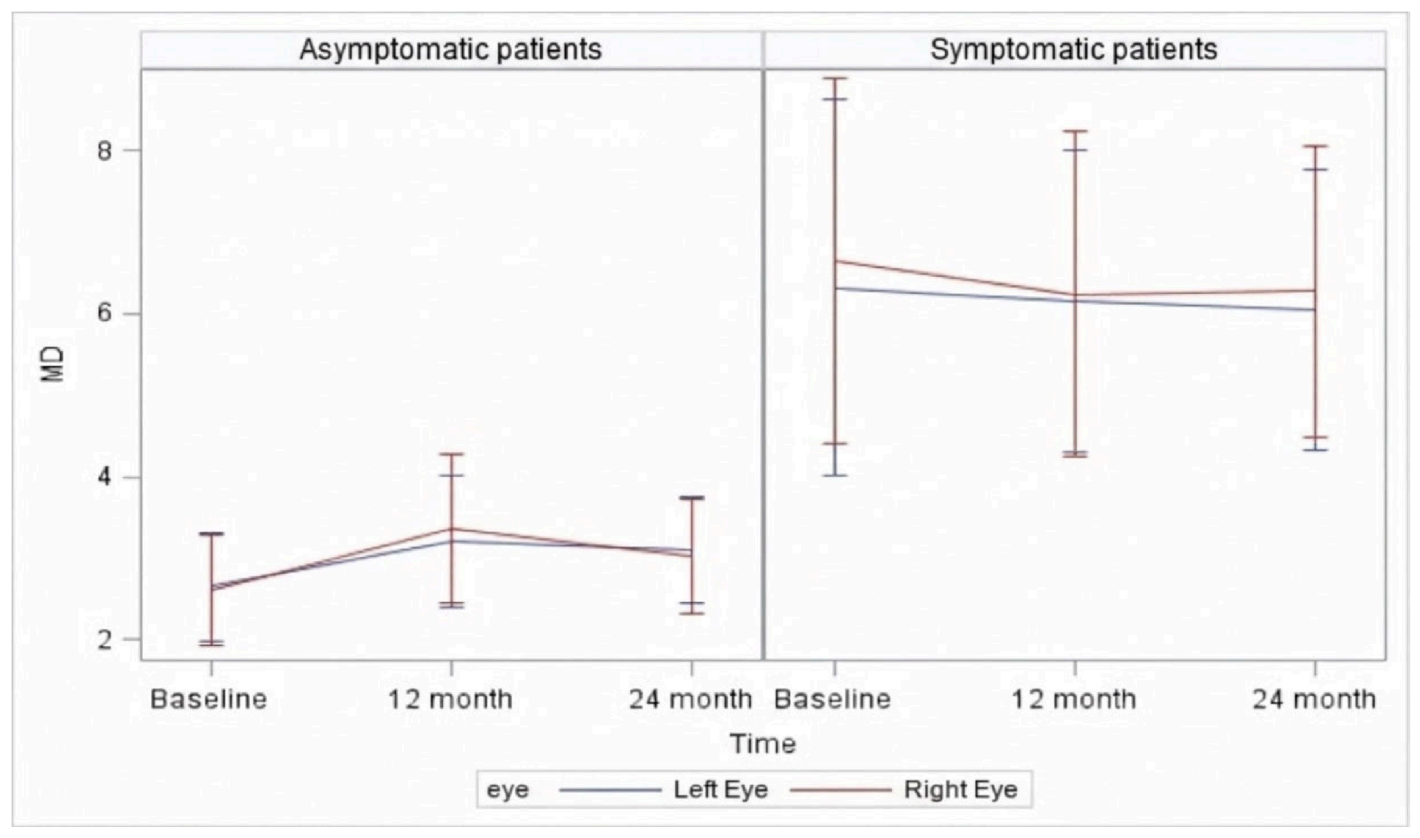
Longitudinal changes in visual field mean defect (MD) over 24 months in the bilateral uveitis group. Error bars represent 95% confidence intervals.

**Figure 6:**
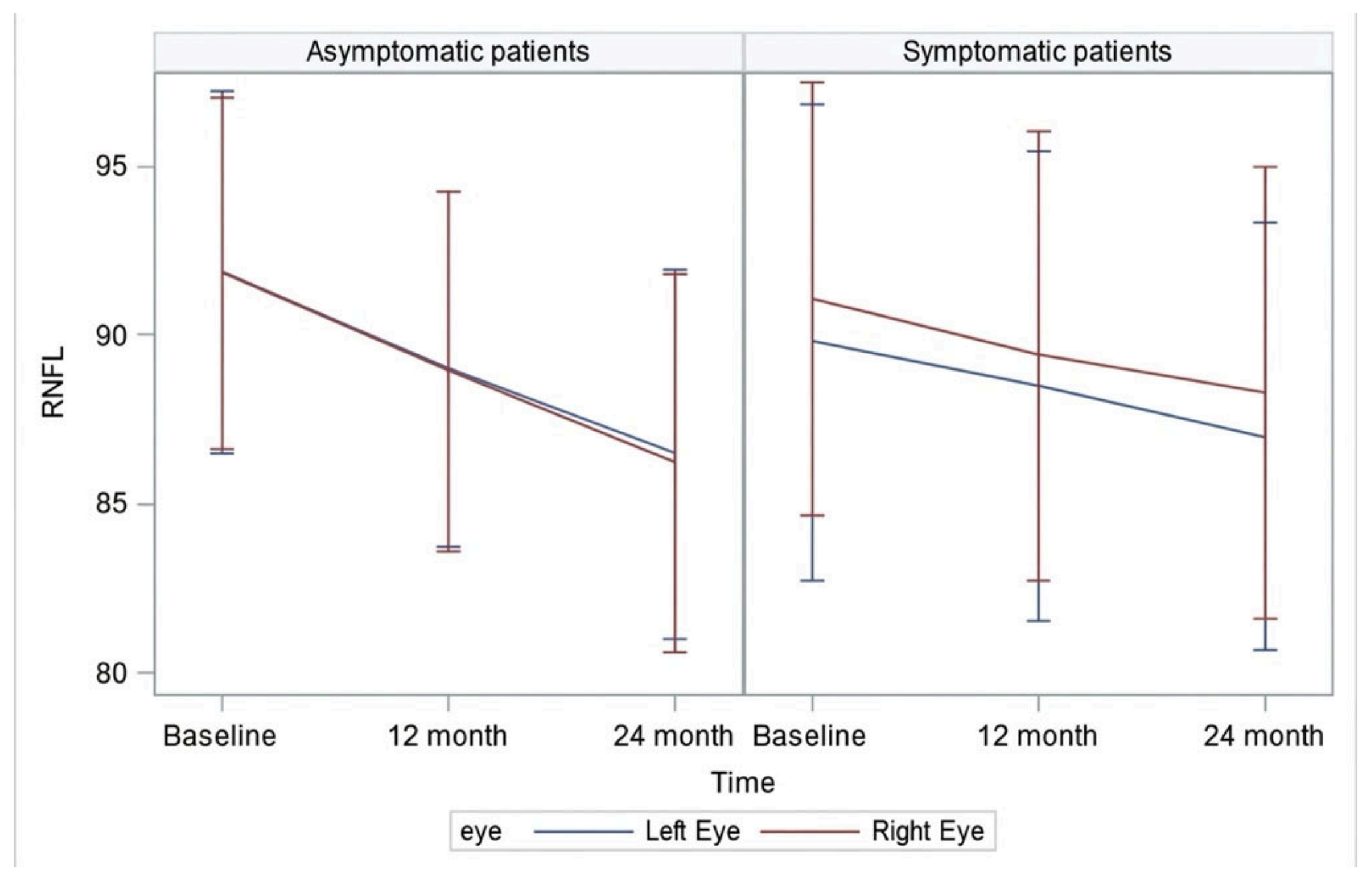
Longitudinal changes in retinal nerve fiber layer (RNFL) thickness over 24 months in the bilateral uveitis group. Error bars represent 95% confidence intervals.

Of the 24 patients with bilateral uveitis, 14 were asymptomatic (12 intermediate, 2 posterior) and 10 symptomatic (7 intermediate, 3 posterior). In the bilateral uveitis group, the linear mixed model showed that symptomatic status was associated with both functional parameters (Table 6). Longitudinal changes in BCVA logMAR, MD and RNFL are shown in Figure 4, Figure 5 and Figure 6.

### 3.6. Annualized Rates of RNFL Loss and Visual Field MD Progression

Longitudinal linear regression analysis revealed distinct differences in the annualized rates of structural neuroretinal thinning (RNFL loss) and functional visual field deterioration (MD progression) between the anterior uveitis group and the bilateral uveitis group over the 24-month study period. The comprehensive annualized progression metrics, slope coefficients, and linear model goodness-of-fit (R^2^) values for all evaluated cohorts are detailed in Table 7.

### 3.7. Sensitivity Analyses

Annualized rates of change were compared between asymptomatic (*n* = 14) and symptomatic (*n* = 10) patients within the bilateral uveitis group, using the mean of both eyes for each patient. pRNFL loss was greater in asymptomatic patients (−2.75 vs. −1.40 µm/year; difference −1.35 µm/year, 95% CI −2.45 to −0.25; Mann–Whitney *p* = 0.033; Welch *t* test *p* = 0.019), whereas the difference in MD was smaller (+0.22 vs. −0.16 dB/year; difference +0.38 dB/year, 95% CI +0.02 to +0.73; *p* = 0.039). No eye showed an increase in pRNFL thickness at any interval, and thinning over the full 24 months was present in all 28 eyes of asymptomatic patients. After exclusion of the four asymptomatic patients with the greatest loss, the mean rate fell to −1.82 µm/year.

Because all three patients with cystoid macular oedema were symptomatic and received intravitreal therapy, the analysis was repeated after their exclusion (*n* = 21). The structural finding was unchanged and marginally more pronounced: −2.38 µm/year (right eye) and −2.24 µm/year (left eye), with the difference between symptomatic and asymptomatic patients remaining similar in direction and magnitude (Welch *t*-test *p* = 0.022; primary Mann–Whitney *U* test *p* = 0.054). The functional finding did not persist. Mean defect in the CME patients improved by −0.70 dB/year (10.82 ± 0.25 to 9.42 ± 0.53 dB). In the remaining seven symptomatic patients it was unchanged (+0.07 dB/year, 95% CI −0.20 to +0.34; *p* = 0.539) and the difference from asymptomatic patients was no longer significant (+0.15 dB/year, 95% CI −0.14 to +0.44; *p* = 0.292). Best-corrected visual acuity was unchanged after exclusion of the CME patients (group mean 0.039 logMAR at baseline, 12 and 24 months).

Because all eight anterior uveitis patients were symptomatic and the bilateral group combined two anatomical entities, the comparison was repeated restricted to intermediate uveitis alone (*n* = 19; 12 asymptomatic, 7 symptomatic), a single anatomical subtype. The direction and magnitude of the structural difference were preserved (−2.62 vs. −1.36 µm/year), though statistical significance again depended on the inferential method (Welch *t*-test *p* = 0.042; primary Mann–Whitney *U* test *p* = 0.060). All three patients with cystoid macular oedema had intermediate uveitis and were retained in this analysis, so the perimetric difference in this subgroup (*p* = 0.047) remains subject to the same caveat as in the full group.

## 4. Discussion

In this retrospective cohort of 32 patients with MS-associated uveitis followed for 24 months, three findings stand out. First, uveitis was identified in 3.8% of MS patients referred for neuro-ophthalmological assessment, and intermediate uveitis was the predominant anatomical type (59.4%), consistent with the existing literature. Second, 43.8% of patients reported no ocular symptoms at any visit, yet visual field defects were present in 84.4% of the cohort despite preserved visual acuity, indicating that a substantial proportion of MS-associated intraocular inflammation is clinically silent but not functionally silent. Third, peripapillary RNFL thickness declined significantly over 24 months in every subgroup examined—including eyes with no history of optic neuritis and the uninflamed fellow eyes of patients with unilateral anterior uveitis—while pRNFL loss was faster in asymptomatic patients; the corresponding difference in mean defect was attributable to resolution of cystoid macular oedema in three treated patients. None of the asymptomatic patients would have been referred on the basis of ocular complaints; they were identified only because ophthalmological examination was performed irrespective of symptoms.

The association between multiple sclerosis (MS) and uveitis has been known for decades. From the available literature, a range of 0.65% to 36.7% has been reported. Such a wide range can be explained by differences in study design, population selection, and diagnostic criteria for uveitis [4,8,15]. In our study, uveitis was found in 32 of 852 patients, which gives a frequency among referred patients of 3.8%. A recent meta-analysis reported a frequency of uveitis among people with MS of 1.1% (95% CI: 0.6–1.7%), with intermediate uveitis being the most common [9]. The relatively low frequency of the disease means that most of the available literature comes from observational studies with limited samples, usually conducted in single centres, which may not fully reflect the actual clinical picture [6]. Common immunological mechanisms are the pathogenetic basis of the connection between MS and uveitis. Genome-wide association studies (GWAS) have identified loci responsible for up to 30% of an individual’s risk of developing MS, many of which overlap with genetic risk factors for intermediate uveitis—particularly the human leukocyte antigen (HLA) II genes classes, including HLA-DR15 and HLA-DR51 [5]. Both MS and intermediate uveitis are associated with the HLA-DRB1*15:01 allele and the rs2104286 A>G polymorphism of the IL-2RA gene, which points to a common genetic background in which T-lymphocytes play an important role—and probably B-lymphocytes [15]. Key effector cells, both in uveitis and relapsing MS, include specific subsets of CD4+ T-helper cells type 1 (Th1) that produce interferon-gamma (IFN-γ) and TNF-alpha (TNF-α) and CD4+ T-cells that produce IL-17 (Th17). The female predominance in our cohort (65.6%) is consistent with the known epidemiology of both MS and MS-associated uveitis. In a large meta-analysis, 72% of MS patients with uveitis were female, with a mean age of 43.5 years [9]. Another meta-analysis reported a mean age of 38.2 years with a female predominance of 67% [6].

The results of this study confirm that intermediate uveitis is the predominant form of uveitis in patients with MS, consistent with the existing literature [16]. We found that intermediate uveitis accounted for 59.4% of cases. Bilateral intermediate uveitis is the most frequently described form in the literature [5,6], and our findings are consistent with this observation. Saboya-Galindo et al. reported that intermediate uveitis was found in 68% of MS-associated uveitis cases [6]. Vaheb et al. similarly noted that intermediate uveitis is the most common form [9]. However, Towler et al. and Schmidt et al. reported anterior uveitis as the most common form, while other studies have described posterior uveitis and panuveitis [3,17,18]. Notably, visual acuity and visual field defect distributions were highly symmetric between the right and left eyes in our cohort, consistent with the predominantly bilateral nature of MS-associated uveitis.

In the majority of reported cases, uveitis was bilateral. Casselman et al. stated that MS-associated uveitis is predominantly bilateral and that intermediate uveitis with vasculitis is the most common form [15]. Jouve et al. found bilateral involvement in 32 of 36 patients [19], and Raja et al. reported bilateral uveitis in 94% of patients [20]. Although the precise mechanism underlying bilateral involvement is not fully understood, it is thought that both MS and uveitis share similar immune-pathogenetic mechanisms, with autoimmunity against non-myelin antigens co-expressed in both the uvea and the CNS [5].

Anterior uveitis (iridocyclitis) was the second-most common type in our series (25.0%), and all patients in this group were symptomatic, presenting with the classic triad of redness, pain, and photophobia. Le Scanff et al. found that anterior uveitis accounted for 28.6% of cases in their series [21], and our finding of 25.0% falls within the reported range of 14.3–28.6% [21,22].

A striking finding in our study was the high proportion of asymptomatic uveitis—63.2% of patients with intermediate uveitis and 43.8% of the total cohort had no ocular symptoms at the time of diagnosis. This high proportion of asymptomatic patients can be explained by the fact that the majority of patients did not have macular involvement. When inflammation affects the pars plana and peripheral vitreous without involving the macula, symptoms may not be present. In most such patients, pain is absent, and there are no visible changes in the anterior segment of the eye, unlike in anterior uveitis. Intermediate uveitis is a slowly progressing and insidious inflammation that may not produce alarming symptoms. Consequently, since there are no changes in the anterior segment such as redness, intermediate uveitis often goes unnoticed by both the patient and the non-ophthalmologist clinician.

Additionally, patients with MS may have subclinical and chronic optic neuritis that can mask the symptoms of intermediate uveitis. The asymptomatic presentation is therefore likely the result of a combination of a painless, slowly progressing inflammatory process affecting both eyes [5,23]. This finding underscores the insidious nature of MS-associated uveitis and highlights the critical importance of routine ophthalmological screening. Recent reviews have emphasised the importance of recognising uveitis as a comorbidity in the routine clinical evaluation and management of MS patients, and that diagnosis by specialists within dedicated clinics is imperative [4]. As this study includes only ophthalmologically examined patients, the proportion of asymptomatic patients could be higher. Namely, due to the absence of symptoms, they are not referred for an ophthalmological evaluation.

The visual acuity data in our study showed that 16 of the 19 (84.2%) patients with intermediate uveitis within the bilateral uveitis group maintained full visual acuity, consistent with the predominantly subclinical course of this uveitis subtype. In contrast, patients with iridocyclitis and posterior uveitis had more heterogeneous visual outcomes, reflecting the symptomatic nature of these conditions. At baseline, only 3 patients with anterior uveitis had reduced visual acuity, and mean logMAR values in this group decreased at the follow-up visits (Table 3), although this change was not statistically significant. Cystoid macular oedema (CME) was present in 3 patients with intermediate uveitis (15.8% of the patients with intermediate uveitis), representing the most significant vision-threatening complication. The Dexamethasone implant was given once and not repeated, so the perimetric improvement recorded at 12 and 24 months reflects sustained resolution of the oedema rather than continuing pharmacological effect. Visual acuity improved in all three patients with cystoid macular oedema following intravitreal therapy (from 0.52–1.00 to 0.30–0.40 logMAR) but did not return to normal, consistent with the absent ellipsoid zone and external limiting membrane at the fovea in all six eyes. This finding strongly correlates with reduced or lost visual acuity [24]. Three patients had decreased visual acuity, central blurring, and impaired colour vision, which are symptoms initially suggestive of acute optic neuritis. But further examination discovered CME in those 3 patients. This distinction is clinically important, as CME can be mistaken for recurrent optic neuritis, the most common visual system manifestation in MS [25]. Therefore, thorough examination, including OCT and visual field testing, is essential to identify the true cause of visual impairment.

An important contribution of this study is the systematic assessment of visual field defects using Octopus perimetry. Visual field abnormalities were present in the majority of patients (84.4%), even in the bilateral uveitis group, where 79.2% had abnormal perimetry despite the high proportion of asymptomatic patients. The most common defect patterns were peripheral depression, peripheral depression with paracentral scotoma and central scotoma. These findings suggest that subclinical visual field loss may be more prevalent than currently appreciated in MS-associated uveitis. The defects may result from multiple mechanisms, including macular oedema, vitreous opacities, retinal vasculitis, and subclinical optic nerve involvement. Although patients with acute optic neuritis were excluded from this study, the high proportion of visual defects located in the intermediate and peripheral zones across all groups may reflect both uveitis-related damage and subclinical MS-related optic nerve pathology. In the three patients with CME, Octopus perimetry demonstrated central scotomas, as it did in the three symptomatic patients with posterior uveitis. Central scotomas—round defects centred on the fixation point—are caused by loss of photoreceptors or ganglion cell axons serving the macula. In contrast, cecocentral scotomas involve a larger number of axons in the papillomacular bundle, indicating optic nerve pathology [26]. Therefore, the distinction between central and cecocentral scotomas is important in differentiating macular disease from optic neuritis, and visual field testing should be performed in addition to OCT to clarify the aetiology of visual impairment [27].

This study demonstrated progressive thinning of the retinal nerve fibre layer in patients in the bilateral uveitis group. Conversely, mean defect did not change significantly over the two years. A dissociation between structural and functional parameters was observed. One possible explanation is that neuroaxonal loss precedes measurable functional change, but this temporal sequence was not demonstrated here. Limited statistical power, the test–retest variability of standard automated perimetry and treatment-related perimetric improvement in the three patients with cystoid macular oedema could all contribute to the absence of a significant mean change. This structural–functional dissociation matches the expanding consensus on the utility of advanced retinal imaging in demyelinating diseases. Pichi et al. reported that retinal macrophage-like cell activation and neuroretinal layer thinning are associated with higher clinical disability and T2 lesion burden on brain MRI [28]. That study was cross-sectional, so it indicates which retinal measures merit assessment rather than establishing an inflammatory mechanism or a rate of thinning applicable to the present cohort. Furthermore, longitudinal data employing a rebaselining concept have confirmed that such retinal layer thinning remains a robust and sensitive biomarker capable of tracking this neuroaxonal loss well below the clinical threshold, registering ongoing tissue decline even in patients demonstrating complete clinical and relapse-free stability [29]. For clinical practice, this implies that evaluation of the visual system should involve both structural and functional assessments; in this patient population, OCT and perimetry provide complementary rather than interchangeable information.

These observations highlight important clinical considerations within this referral context. Because almost half of the patients in this cohort reported no ocular symptoms at any visit, and because none of the asymptomatic patients would have been referred on the basis of complaints alone, relying strictly on a symptom-driven referral strategy may overlook a substantial proportion of MS-associated uveitis cases. While our data from a selected referral cohort cannot establish the necessity, optimal timing, or cost-effectiveness of a universal screening programme, the frequent presence of subclinical inflammation suggests that considering an early, comprehensive ophthalmological assessment—comprising slit-lamp biomicroscopy, dilated fundoscopy, OCT and perimetry—could be beneficial in identifying the intermediate uveitis and peripheral retinal vasculitis that dominated the asymptomatic subgroup here. Another closely related consideration concerns attribution: in three patients, cystoid macular oedema presented with reduced acuity, central blurring and impaired colour vision, a constellation that would ordinarily suggest recurrent optic neuritis. Distinguishing the two requires macular OCT and perimetry rather than history alone, and the distinction is therapeutically consequential. Conversely, and consistent with current recommendations, patients presenting with idiopathic intermediate uveitis warrant neurological assessment for demyelinating disease [9,13].

In this cohort the rate of pRNFL loss was higher than rates reported in MS eyes without optic neuritis and in healthy controls [10]. Several considerations apply to this comparison. First, to our knowledge no published cohort combines multiple sclerosis, absence of optic neuritis and concurrent intraocular inflammation, so the available reference values derive from non-inflamed eyes and may not be an appropriate comparator. Second, individual changes were modest relative to the reported measurement variability of the device [30]; the observation rests on the direction and distribution of change rather than on its magnitude, as no eye showed an increase in pRNFL thickness at any interval and thinning over 24 months was present in all 28 eyes of asymptomatic patients. In an exploratory comparison within the bilateral uveitis group, in which all patients had bilateral intermediate or posterior uveitis and uveitis type was entered as a fixed factor in the model, pRNFL loss was greater in asymptomatic than in symptomatic patients (−2.75 vs. −1.40 µm/year; difference −1.35 µm/year, 95% CI −2.45 to −0.25; Mann–Whitney *p* = 0.033, Welch *p* = 0.019). Loss exceeding 5 µm over 24 months occurred in 5 of 14 asymptomatic and in none of the 10 symptomatic patients. After exclusion of the four asymptomatic patients with the greatest loss the mean rate was −1.82 µm/year, above the symptomatic rate, with no significant difference in the reduced sample (*p* = 0.126). Two sensitivity analyses were performed (Section 3.7, Table 8). After exclusion of the three patients with cystoid macular oedema, and in the analysis restricted to intermediate uveitis, the estimated difference was similar in direction and magnitude to that in the full group (−1.32 and −1.27 vs. −1.35 µm/year), but statistical significance depended on the test applied (Mann–Whitney *p* = 0.054 and *p* = 0.060; Welch *p* = 0.022 and *p* = 0.042). The perimetric difference did not persist: mean defect improved by −0.70 dB/year in the three patients with cystoid macular oedema and was unchanged in the remaining seven symptomatic patients (+0.07 dB/year, *p* = 0.539), with no significant difference from asymptomatic patients (*p* = 0.292). The perimetric difference between symptomatic and asymptomatic patients was no longer evident after exclusion of these three patients and therefore appears to be driven largely by their functional improvement rather than by symptomatic status; with three patients this cannot be established as a causal effect. A third consideration is subclinical inflammatory peripapillary oedema, which could produce apparent thinning as it resolves. Asymptomatic patients showed greater loss than symptomatic patients, which is not the pattern this mechanism would predict. Symptomatic status is, however, not a measure of inflammatory activity, and resolving subclinical peripapillary swelling cannot be excluded on this basis. In the anterior uveitis group, where inflammation was unilateral, no difference in pRNFL loss was detected between affected and fellow eyes over 24 months (1.38 ± 0.74 vs. 2.00 ± 0.76 µm; Wilcoxon signed-rank *p* = 0.25). With eight patients this comparison has very low power, and the absence of a detected difference neither establishes equivalence between the two eyes nor excludes a contribution of local inflammation. The comparison between symptomatic and asymptomatic patients was internally controlled, using the same device, protocol, centre and study period. It was nevertheless exploratory: the subgroups were small, several related comparisons were performed, no adjustment for multiplicity was applied, and significance in the restricted samples was test dependent. The finding requires confirmation in larger prospective cohorts with concurrent analysis of macular ganglion cell layer thickness.

This study has several limitations. The sample size is relatively small (n = 32), which limits statistical power and generalizability. The retrospective design introduces potential selection and information bias. Visual field defect categories were concordant between eyes in all bilateral cases, so the effective sample size approximates the number of patients rather than the number of eyes, and the precision of the estimates should be interpreted accordingly. The annualized rates in Table 7 are derived by fitting linear regression to group mean values at three time points and therefore describe the group trend without incorporating between-patient variability; the comparisons in Section 3.7 used patient-level rates and are not subject to this limitation. Because a substantial proportion of patients were asymptomatic and treatment was not standardised, the effect of therapy on visual function could not be assessed beyond the three patients with cystoid macular oedema. The temporal relationship between uveitis onset and MS diagnosis was not systematically analysed; in a recent meta-analysis, MS development preceded uveitis in 59% of cases, while uveitis preceded MS in 38% [6]. Finally, the visual field defects observed may have multifactorial aetiologies, and distinguishing uveitis-related from MS-related loss remains challenging. No healthy control group was included. Comparisons relied on the non-inflamed fellow eyes of the anterior uveitis group and on the device normative database; as the fellow eyes themselves showed perimetric abnormalities, they represent an internal reference for the effect of inflammation rather than a normal control. Disease-modifying therapy status was undocumented in the referral records for 15 of 32 patients (47%) and could therefore not be entered into the statistical model. As disease-modifying therapy may influence both inflammatory activity and the rate of neuroaxonal loss, its contribution to the observed difference between symptomatic and asymptomatic patients cannot be excluded. Symptomatic and asymptomatic patients were not matched on any variable. Comparability was addressed only by restriction to the bilateral uveitis group and, in a sensitivity analysis, to intermediate uveitis alone, and by entering uveitis type as a fixed factor in the model. A non-significant subtype term does not exclude residual confounding by anatomical phenotype, particularly given the small number of posterior uveitis cases. Future prospective studies with larger cohorts, standardised longitudinal perimetry and correlation with OCT structural data are warranted.

## 5. Conclusions

In our study, we found that intermediate uveitis is the most common form of uveitis in patients with multiple sclerosis. A significant number of patients were asymptomatic, yet visual field defects were common despite generally preserved visual acuity. The nerve fibre layer showed thinning over a 24-month period in both groups. As all patients had both multiple sclerosis and uveitis and no comparison group of MS patients without uveitis was available, the origin of this thinning cannot be attributed to intraocular inflammation rather than to diffuse MS-related neurodegeneration. This suggests that patients with multiple sclerosis should undergo an ophthalmological examination, regardless of whether they report any ocular symptoms. Conversely, patients with idiopathic uveitis should be evaluated for multiple sclerosis. An interdisciplinary approach to the care of these patients is crucial.

## Figures and Tables

**Table 1:** Distribution of patients by uveitis type, age, symptomatic status and MS duration.

Group	*n*	Mean Age ± SD (Years)	Age Range (Years)	Symptomatic	Asymptomatic	Bilateral	MS Duration (Years)
Anterior uveitis	8	36.1 ± 6.4	28–46	8 (100%)	0 (0%)	0 (0%)	5.4 ± 2.5
Intermediate uveitis	19	34.3 ± 6.2	22–45	7 (36.8%)	12 (63.2%)	19 (100%)	4.6 ± 3.4
Posterior uveitis	5	33.2 ± 5.0	28–39	3 (60%)	2 (40%)	5 (100%)	4.4 ± 2.6
Total	32	34.59 ± 6.01	22–46	18 (56.3%)	14 (43.8%)	24 (75%)	4.8 ± 3.0

**Table 2:** Ocular and disease-modifying treatment by group. Ocular treatment categories are not mutually exclusive; patients could receive more than one treatment concurrently. Disease-modifying therapy categories are mutually exclusive and sum to the group total.

Treatment	Anterior Uveitis Group (*n* = 8)	Bilateral Uveitis Group, Asymptomatic (*n* = 14)	Bilateral Uveitis Group, Symptomatic (*n* = 10)
Topical corticosteroid	8 (100%)	14 (100%)	10 (100%)
Mydriatic	8 (100%)	0	0
Intravitreal therapy (0.7 mg dexamethasone intravitreal implant)	0	0	3 (30%)
Systemic corticosteroid therapy	0	2 (14%)	3 (30%)
MS disease-modifying therapy			
Receiving DMT	3 (37.5%)	5 (35.7%)	4 (40%)
Not receiving DMT	2 (25.0%)	3 (21.4%)	0
Status not documented	3 (37.5%)	6 (42.9%)	6 (60%)

**Table 3:** Visual acuity in both groups.

	Anterior Uveitis Group		Bilateral Uveitis Group	
BCVA (logMAR)	Affected Eye	Fellow Eye	Right Eye	Left Eye
At baseline	0.05 ± 0.07	0.00 ± 0.00	0.13 ± 0.26	0.08 ± 0.20
After 12 Months	0.02 ± 0.04	0.00 ± 0.00	0.08 ± 0.14	0.05 ± 0.11
After 24 Months	0.01 ± 0.02	0.00 ± 0.00	0.08 ± 0.14	0.05 ± 0.11

**Table 4:** Distribution of patients across all groups according to Visual field defects.

	Bilateral Uveitis Group						Anterior Uveitis Group					
	Baseline		12 Months		24 Months		Baseline		12 Months		24 Months	
	RE	LE	RE	LE	RE	LE	AE	FE	AE	FE	AE	FE
Normal finding	5	5	4	4	4	4	0	0	0	0	0	0
Central scotoma	6	6	6	6	6	6	0	0	0	0	0	0
Peripheral depression and paracentral scotoma	2	2	2	2	2	2	4	0	2	0	1	0
Nerve fibre bundle defect	3	3	4	4	5	5	2	6	4	6	5	6
Sectoral defect	3	3	4	4	3	3	1	1	1	1	1	1
Peripheral depression	5	5	4	4	4	4	1	1	1	1	1	1

Note: AE: affected eye; FE: non-inflamed fellow eye; RE: right eye; LE: left eye.

**Table 5:** Results of Linear Mixed Model for anterior uveitis group.

Dependent Variable	BCVA			MD			RNFL		
Effect	BCVA F	BCVAP-Value	BCVA Post Hoc	MD F	MD *p*-Value	MD Post Hoc	RNFL F	RNFL *p*-Value	RNFL Post Hoc
Eye	3.34	0.0902	-	24.10	0.0014	AE vs. FE	2.91	0.1065	-
Time	2.53	0.0989	-	3.64	0.0394	B vs. 24	22.75	<0.0001	B vs. 12; B vs. 24
Eye × time	2.53	0.0989	-	7.04	0.0033	AE: B vs. 24	0.91	0.4151	-

Note: AE: affected eye; FE: fellow eye; B: baseline; 12: 12 months; 24: 24 months. Significant pairwise differences (Tukey–Kramer adjusted) are listed in the post hoc column. Identical F statistics for the time main effect and the eye × time interaction arise because BCVA was invariant (0.00 logMAR at all visits) in the fellow eyes, so the two contrasts differ only by a scale factor. The eye × time interaction reflects a decrease in mean defect in affected eyes (−0.37 dB/year) with no change in fellow eyes (+0.06 dB/year).

**Table 6:** Results of Linear Mixed Model for the bilateral uveitis group.

Dependent Variable	BCVA			MD			RNFL		
Effect	F	*p*-Value	Post Hoc	F	*p*-Value	Post Hoc	F	*p*-Value	Post Hoc
S-A	10.76	0.0036	S vs. A	15.01	0.0009	S vs. A	0.01	0.9354	-
Eye	7.19	0.0130	RE vs. LE	1.16	0.2906	-	1.24	0.2780	-
Time	9.30	0.0002	B vs. 12; B vs. 24	1.91	0.1543	-	74.01	<0.0001	B vs. 12; B vs. 24
Uveitis type	0.04	0.8401	-	0.01	0.9262	-	0.14	0.7130	-
Time × S-A	9.30	0.0002	-	10.90	<0.0001	-	7.82	0.0008	-
Time × Eye	0.40	0.6728	-	0.06	0.9388	-	0.08	0.9262	-
Eye × S-A	7.57	0.0109	-	1.09	0.3047	-	2.10	0.1632	-

Note: RE: right eye; LE: left eye; S: symptomatic; A: asymptomatic; B: baseline; 12: 12 months; 24: 24 months. Significant pairwise differences (Tukey–Kramer adjusted) are listed in the post hoc column. Identical F statistics for the time main effect and the time × S-A interaction arise because BCVA was invariant (0.000 logMAR at all visits) in the asymptomatic group, so the two contrasts differ only by a scale factor.

**Table 7:** Annualized progression metrics for RNFL thickness and visual field MD.

Uveitis Group	Eye	Annualized RNFL Change (μm/Year)	RNFL Model Fit (R^2^)	Annualized MD Change (dB/Year)	MD Model Fit (R^2^)	95% CI MD Slope
Anterior uveitis Group (*n* = 8)	Affected eye	−0.69	0.818	−0.37	0.995	−0.57 to −0.17
	Fellow Eye	−1.00	0.980	+0.06	0.987	0.00 to +0.13
Bilateral uveitis group (*n* = 24)	Right Eye	−2.23	0.999	+0.04	0.104	−0.13 to +0.22
	Left Eye	−2.15	0.998	+0.08	0.323	−0.10 to +0.25

Annualized rates were obtained by linear regression fitted to the group mean values at baseline, 12 and 24 months; R^2^ describes the fit of that linear trend to three aggregate time points. These values summarise the group trend and do not incorporate between-patient variability. Confidence intervals for between-group comparisons are reported in Section 3.7, where patient-level annualized rates were the unit of analysis.

**Table 8:** Difference in annualized pRNFL rate of change between asymptomatic and symptomatic patients.

Analysis	n (Asympt./Sympt.)	Difference (µm/Year)	95% CI	Mann–Whitney *p* (Primary)	Welch *p*
Bilateral uveitis, all	14/10	−1.35	−2.45 to −0.25	0.033	0.019
Bilateral uveitis, CME excluded	14/7	−1.32	−2.43 to −0.22	0.054	0.022
Intermediate uveitis only	12/7	−1.27	−2.48 to −0.05	0.060	0.042

## Data Availability

The data presented in this study are available on request from the corresponding author. The data are not publicly available due to privacy and other ethical restrictions.

## Abbreviations

The following abbreviations are used in this manuscript:

AR(1)	first-order autoregressive
BCVA	best-corrected visual acuity
CI	confidence interval
CME	cystoid macular oedema
CNS	central nervous system
DMT	disease-modifying therapy
EDSS	Expanded Disability Status Scale
FA	fluorescein angiography
GWAS	genome-wide association study
HLA	human leukocyte antigen
IFN-γ	interferon-gamma
IL-2RA	interleukin-2 receptor alpha
logMAR	logarithm of the minimum angle of resolution
MD	mean defect
MRI	magnetic resonance imaging
MS	multiple sclerosis
OCT	optical coherence tomography
pRNFL	peripapillary retinal nerve fibre layer
REML	restricted maximum likelihood
RF	reliability factor
SD	standard deviation
STROBE	Strengthening the Reporting of Observational Studies in Epidemiology
SUN	Standardization of Uveitis Nomenclature
Th1	T-helper cell type 1
Th17	interleukin-17-producing T-helper cell
TNF-α	tumour necrosis factor alpha
